# The Applicability of Essential Oils in Different Stages of Production of Animal-Based Foods

**DOI:** 10.3390/molecules26133798

**Published:** 2021-06-22

**Authors:** Weronika Mucha, Dorota Witkowska

**Affiliations:** Department of Animal and Environmental Hygiene, Faculty of Animal Bioengineering, University of Warmia and Mazury in Olsztyn, 5 Oczapowski Street, 10-719 Olsztyn, Poland; weronika.mucha@student.uwm.edu.pl

**Keywords:** essential oil, animal-based foods, from stable to table, public health safety, food preservation, green pesticides

## Abstract

Essential oils (EOs) have been used for centuries, and interest in these compounds has been revived in recent years. Due to their unique chemical composition as well as antimicrobial, immunostimulatory, anti-inflammatory and antioxidant properties, EOs are used in pharmacology, cosmetology and, increasingly, in animal breeding and rearing, and processing of animal raw materials. Essential oils have become a natural alternative to preservatives, taste enhancers and, most importantly, antibiotics, because the European Union banned the use of antibiotics in metaphylaxis in animal husbandry in 2006. In the animal production chain, EOs are used mainly as feed additives to improve feed palatability and increase feed intake, improve animal resistance and health status, and to prevent and treat diseases. Recent research indicates that EOs can also be applied to sanitize poultry houses, and they can be used as biopesticides in organic farming. Essential oils effectively preserve meat and milk and, consequently, improve the safety, hygiene and quality of animal-based foods. Novel technologies such as encapsulation may increase the bioavailability of EOs and their application in the production of food and feed additives.

## 1. Introduction

Essential oils (EOs) are natural plant products with a rich chemical composition and diverse biological properties [1]. These mixtures of volatile compounds are produced by living organisms and are isolated by physical methods (pressing and distillation) from whole plants or selected plant parts [2]. The composition of EOs is determined by the plant family, genus or species, as well as the growing conditions, season of harvest and the geographic origin of the raw materials [3,4].

Essential oils have been used by humans for millennia. The oldest known evidence of EO isolation from plants dates back 5000 years. Aromatic substances were also popular in ancient Rome, Greece, the Middle East and the Far East. The therapeutic and repellent properties of aromatic plants were recognized in Europe, and research into EOs had already begun during the Renaissance period [5]. Essential oil components were identified in the 19th century, which contributed to the development of the pharmaceutical industry. However, further scientific inquiry into EOs was halted after antibiotics were discovered in the 20th century as the most effective treatment for bacterial infections [6]. The interest in EOs has been revived in recent years. One of the reasons for the above is the growing antimicrobial resistance of many bacteria as well as concerns regarding antibiotic use in animal diets, which contributes to the emergence of antibiotic-resistant bacteria and poses a serious health risk for humans [7]. To address these issues, in 2006, the European Union banned the use of antibiotic growth promoters in animal nutrition [8]. This ban prompted the search for alternative antimicrobials, including herbs and EOs [9]. Additionally, scientists have turned their attention to plants, herbs and their derivatives, due to changes in consumer preferences and the growing interest in natural or low-processed foods without chemical additives [10]. One of the relatively new and still current directions in the development of the food market is the concept of a “clean label”, which is attributed to food production and processing based on natural methods, in a clean and safe environment, practically without chemical additives and fertilizers, synthetic plant protection products, or antibiotics, maintaining a complete ban on growing genetically modified crops and feeding animals with feeds that are derived from such crops [11,12]. Although the price of organic food is even up to 50% higher, consumers are willing to pay more for better quality and safer products [12].

In view of the above, the aim of this review article was to analyze new trends in animal nutrition and food processing, and the effectiveness of EOs applied in different stages of the animal-based food chain (stable-to-table) to improve the hygiene and quality of the end products. The literature research was based mostly on articles from the last decade.

## 2. Popular Essential Oils

Essential oils are widely used in human medicine. Their biocidal and anti-inflammatory properties have been harnessed by the pharmaceutical industry. Essential oils are applied in the treatment of respiratory disorders and skin diseases; they are used as analgesics and as warming agents in massage therapy. Plant oils can be used alone, and they can be added to pharmaceutical products [13].

Essential oils derived from the plants of the families Alliaceae (onion), Apiaceae (celery), Asteraceae (aster), Lamiaceae (oregano, thyme, lavender, peppermint, sage oils), Lauraceae (cinnamon oil), Liliaceae (garlic oil), Myrtaceae (tea tree oil), Poaceae (grass) and Rutaceae (rue) have been well-researched on account of their therapeutic properties and industrial applications (Table 1). Oregano, cinnamon, garlic, thyme, black pepper, lavender, mint, sage and tee tree EOs are used extensively around the world due to their specific chemical compositions [4,6].

### Biocidal Properties of Essential Oils

The bacteria listed in Table 2 cause serious infectious diseases in various farm animal species [CVM]. The treatment of bacterial diseases involves the use of antibiotics, which has a major impact on the quality of animal-based foods [32,33,34]. Bacterial species such as *Salmonella typhimurium*, *Listeria monocytogenes*, *Yersinia enterocolitica*, *Escherichia coli*, *Staphylococcus aureus* and *Clostridium botulinum* pose a serious biological risk in animal-based foods, and they can lead to food poisoning [35].

The results of studies investigating the sensitivity of animal pathogenic bacteria to selected EOs are presented in Table 2. Cinnamon EO was found to inhibit the growth of most of the analyzed bacterial pathogens. The antibacterial activity of cinnamon EO against *Clostridium perfringens*, *C. botulinum*, *S. aureus*, *Enterococcus faecalis*, *S. typhimurium, E. coli*, *Y. enterocolitica*, *Klebsiella pneumoniae*, *L. monocytogenes, Proteus vulgaris* and *Pseudomonas aeruginosa* was confirmed in the highest number of replicates [3,17,29,36,37]. Oregano and thyme EOs were also found to possess biocidal activity against most of the investigated bacteria. The effectiveness of the remaining oils (listed in the Table 2) was also high, although these oils exerted more selective effects. It should be noted that *E. coli*, *K. pneumoniae*, *P. aeruginosa* and *S. aureus* were targeted by all of the analyzed EOs. In turn, only a few EOs were effective in inhibiting the growth of *B. subtilis*, *C. coli*, *C. botulinum*, *S. epidermidis* and *Y. enterocolitica*, which could be attributed to the fact that these bacterial species were far less frequently investigated [3,15,17,20,23,29,36,37,38,39].

The growth of bacteria was effectively inhibited by the minimum inhibitory concentration (MIC) of EOs, ranging from 0.12 to >2.0 vol% and 0.25–5.0 µL/mL (Table 2) [20,36,38,40]. Oregano EO was the most potent inhibitor of *E. coli*, *K. pneumoniae*, *S. typhimurium* and S. *aureus* (0.12 vol%). Similarly, thyme EO effectively inhibited the proliferation of *E. coli* (0.12 vol%), *K. pneumoniae* and *S. aureus* (0.25 vol%), whereas the activity of S. *typhimurium* was suppressed by thyme EO at MIC > 0.2 vol% [20,36,38]. The highest concentrations of the analyzed EOs (>2.0 vol%) were required to inhibit the proliferation of *P. aeruginosa* [20,38]. The proliferation of *L. monocytogenes* was most effectively inhibited by sage (MIC = 2.0 µL/mL) and thyme (MIC = 3.0–5.0 µL/mL) EOs. The activity of *Y. enterocolitica* was suppressed by thyme EO at MIC of 1 µL/mL [40]. In analyses of *E*. *faecalis*, the lowest MIC values were reported for oregano (0.25 µL/mL), and thyme EOs (0.5 µL/mL) and the highest for lavender EO (>2.0 µL/mL) [20,38].

The microscopic fungi listed in Table 3 also pose a significant problem in the animal production chain. Fungal pathogens cause livestock and plant diseases, and they contribute to food spoilage. These pathogens cause infections of the skin and bodily organs. Mycotoxins, namely the metabolites of filamentous fungi, also pose serious health risks. They are responsible for diseases known as mycotoxicosis, associated with damage to internal organs and skin tissue, which are caused by the consumption of contaminated food, including cereals and animal-based products. After ingestion, mycotoxins are chemically transformed, and they reach muscle tissues, eggs and milk [41].

The results of studies evaluating the inhibitory effects of selected EOs against molds and yeast-like fungi are presented in Table 3 [15,23,29,37,38,41,42]. Tea tree, thyme, lavender and sage EOs inhibited the growth of most of the evaluated fungi, including pathogenic molds and yeasts such as *Aspergillus flavus*, *Aspergillus fumigatus*, *Aspergillus niger*, *Candida albicans*, *Cryptococcus neoformans*, *Microsporum canis*, *Trichophyton mentagrophytes* and *Trichophyton rubrum* [15,29,41]. These findings indicate that C. *albicans* is the most frequently analyzed fungus which is also least resistant to EOs [29,37,38,41,42].

The growth of fungi was effectively inhibited by the MIC of EOs ranging from 0.12 to >2.0 vol%, and the effective MIC values were significantly lower than those required to suppress bacterial growth. Thyme and oregano EOs suppressed the growth of the highest number of fungal species at MIC 0.12–0.5 vol%, sage at MIC 0.12–1.0 vol% and lavender at MIC 0.25–1.0 vol% [43,44,45].

## 3. Feed and Water Supplementation with Essential Oils

**Figure 1:**
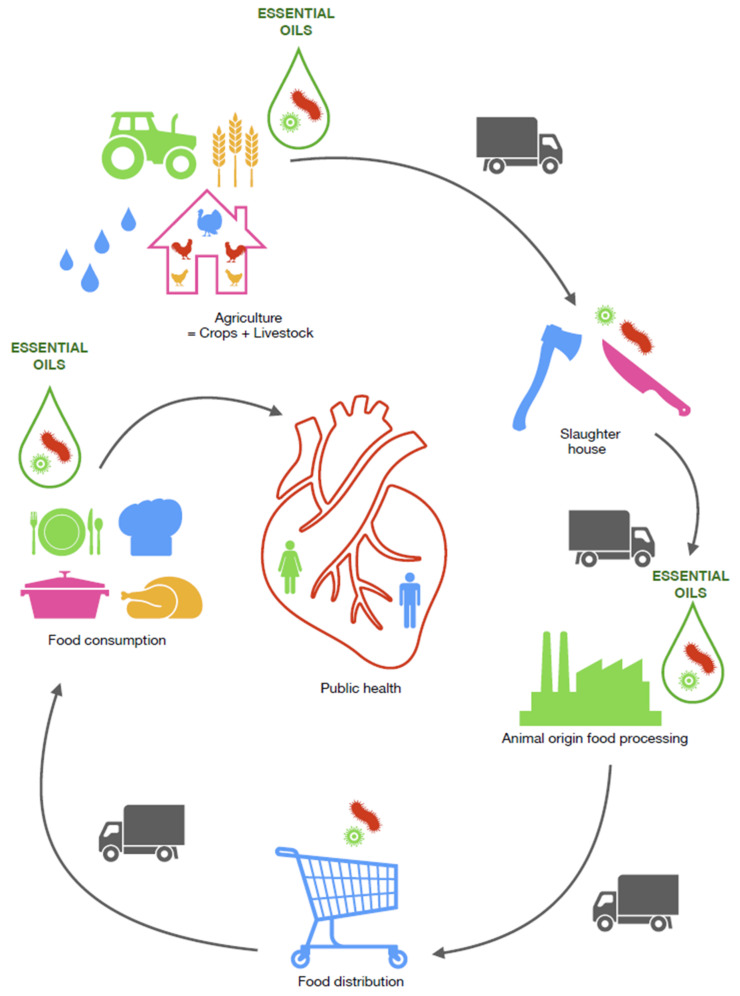
Possibilities of using essential oils in animal production “from stable to table”.

The benefits of EOs were recognized much later in animal breeding, livestock farming and in the production of animal-based foods (Figure 1, Table 4, Table 5) than in human medicine. Essential oils are added to feed and water as taste enhancers, and they are also administered to boost immunity and improve the performance of poultry [16,21].

Since in vitro studies (cited above) have confirmed the antimicrobial efficacy of EOs, they can be used as natural growth promoters in livestock nutrition, in particular in poultry and pig farming [6]. Essential oils enhance the flavor and palatability of feeds, in particular those deficient in these attributes, which improves feed intake. However, EOs should be dosed with caution due to their highly intense aroma, which could compromise feed intake [6,64].

Due to their unique properties, EOs stimulate the secretion of digestive enzymes, affect metabolism and gut microbiota, and improve feed utilization, nutrient digestibility and availability [6]. The effectiveness of herbal supplements is determined by the dose and the content of the main active ingredients [65].

The results of different studies analyzing the effects of dietary supplementation with EOs or their active ingredients on the health status and performance of animals are presented by many authors [47,48,49,50,51,52,53,54,55,56,57,58,59,60,61,62,63].

Essential oils are increasingly used in the prevention and treatment of animal diseases, mostly in monogastric animals, i.e., poultry and pigs [6]. Nehme et al. [34] recognized the immunomodulatory molecules of EOs as a potential therapeutic option in ruminant and monogastric husbandries. In poultry farming, EOs play the role of natural coccidiostats which alleviate the symptoms of bowel diseases and reduce the passage of coccidia oocysts in animal feces. Due to their bactericidal properties, EOs are also applied in pig farms to prevent diarrhea in piglets. Essential oils have been found to boost immunity in livestock. Porcine diets supplemented with EOs enhance the immune response of piglets at weaning [6,32]. In fish diets, EOs may promote local intestinal immunity through the impact on the host-microbial co-metabolism [63]. The antimicrobial activities of EOs can also affect ruminal fermentation [50]. Reduced methanogenesis [66] and nitrogen excretion, and improved digestion were observed in ruminants [32].

According to Frankič et al. [47], EOs (including sage, peppermint and garlic EOs) increase the secretion of fatty acids, bile and digestive enzymes, and exert a positive influence on digestive processes. The mentioned authors report that a mixture of carvacrol, cinnamaldehyde and paprika oleoresin has antioxidant properties, and effectively protects lymphocytes in pig blood against oxidation [47]. Carvacrol isolated from *Origanum* spp. and cinnamic aldehyde extracted from *Cinnamomum* spp. exerted beneficial probiotic effects by increasing the counts of lactic acid bacteria in the porcine digestive tract [48]. Thyme oil improved the intestinal barrier, which protects, e.g., against the passage of toxic substances from poultry feed. The use of carvacrol in feeds may have a positive effect on intestinal morphology, such as increasing the length of poultry intestinal villi [67]. Fish diet supplementation with a blend of microencapsulated garlic, carvacrol, and thymol EOs modulated the intestine transcriptional immune profile and improved microbiota composition [63]. Bölükbaşı et al. [51] reported an improvement in the feed conversion ratio, a decrease in serum cholesterol and triglyceride levels, an increase in egg weight and eggshell proportion in laying hens whose diets were supplemented with thyme, sage and rosemary EOs relative to the control group. The above authors did not observe significant changes in egg white proportion, but reported a decrease in egg yolk proportion in comparison with control eggs [51]. The supplementation of layer hen diets with oregano EO had no significant effect on the feed conversion ratio, feed consumption or egg weight [52]. In a study by Lee et al. [53], carvacrol decreased body weight gains, feed intake and feed efficiency in broilers, whereas thymol did not affect these parameters. In addition, carvacrol reduced triglycerides and phospholipids, but any impact of thymol has been demonstrated. Both carvacrol and thymol had no effect on the presence of total and HDL cholesterol in the plasma [53]. In turn, Denli et al. [62] found that thymol increased body weight gains, feed intake and feed conversion in quails. Compared to the control group, the group fed with thyme oil forage had a higher carcass weight and a lower weight and percentage of abdominal fat. Tiihonen et al. [56] observed higher body weight gains and higher counts of *Lactobacillus* and *E. coli* in the cecum of broiler chickens administered a blend of EOs containing thymol and cinnamic aldehyde. Cetin et al. [59] obtained similar results when using volatile oil mixtures of oregano, rosemary and fennel in poultry diets. Cross et al. [60] reported that thyme and yarrow EOs increased body weight gains, and thyme EO improved feed intake in broilers, but none of the examined EOs exerted probiotic effects. In a study by Jamroz et al. [55], the supplementation of animal diets with extracts (100 mg/kg) containing active ingredients found in thyme and oregano EOs (carvacrol) and cinnamon EO (cinnamic aldehyde) improved the feed conversion ratio by 3.9%. The addition of this mixture resulted in a stronger inhibition of *E. coli* and an increase of *Lactobacillus* spp., as well as the weight of breast muscles. A study by Jerzsele et al. [54] revealed broilers’ resistance to necrotic enteritis (NE), an increase in villus length and the villus length/crypt depth ratio as well as satisfactory performance parameters after ginger oil and carvacrol treatment.

Lippens et al. [68] supplemented broiler chicken diets with a blend of cinnamon, thyme, oregano, plant extracts and organic acids. The performance of these broilers was compared with the control group and birds receiving antibiotic-supplemented feed. In the group administered EOs, the feed conversion ratio was 2.9% lower than in the control group and 0.4% lower than in the antibiotic-supplemented group. In contrast, Hashemipour et al. [61] reported an improvement in the performance of broiler chickens in response to the dietary supplementation with thymol and carvacrol. In addition, the above authors noted positive effects exerted by antioxidant and digestive enzymes, and an enhanced immune response of birds. Similar results (immunomodulatory effects and improved performance) were reported by Awaad et al. [57,58] in an experiment involving broilers receiving peppermint and eucalyptus EOs in water. De Souza et al. [49] found that a blend of natural clove oil and protective additives such as eugenol, thymol and vanillin in heifer diets improved their performance (body weight gains and feed conversion efficiency). Rosemary EO applied alone tended to decrease heifers’ performance, but it positively affected performance parameters when used in a blend of EOs.

Giannenas et al. [65] also noted that in some studies, a specific EO or a blend of EOs did not improve body weight gains, feed intake or the feed conversion ratio. Tekkipe et al. [50] investigated the effects of an EO product (containing eugenol and cinnamaldehyde) on ruminal fermentation, digestibility, and the performance of lactating dairy cows. The cited authors observed only minor effects of the tested EO product on ruminal fermentation and the productivity of lactating dairy cows but found a tendency towards a consistent increase in total-tract NDF digestibility. It should be noted that the analyzed EO product increased cumulative ammonia emission from manure.

Undoubtedly, EOs added to animal diets and water provide many benefits, however, their use is also associated with certain problems and limitations (Table 4). According to many authors [55,56,58,62,69] the efficacy of herbal oil extracts used as feed additives may vary widely depending on their botanical origin, climate, harvest period, methods of extraction, drying and storage, thus leading to inconsistency in the reported findings. The discrepancies in research results concerning the addition of EOs to livestock diets on performance parameters [51,56,61] may be due to the above differences. A strong taste and smell of some Eos, such as carvacrol [47], may negatively affect feed intake by modulating appetite [53]. Frankič et al. [47] demonstrated that EOs can have an adverse effect on gut microbiota, cause allergies and suppress feed intake, and they can also accumulate in tissues. In a study by de Souza et al. [49] rosemary EO applied alone tended to decrease animal performance. Stevanović et al. [70] stressed the fact that the biological effects of EOs are further influenced by the interactions between phytochemicals and their bioavailability in the gastrointestinal tract of animals.

## 4. Fumigation of Animal Houses with Essential Oils

According to a limited number of studies, selected EOs can be effectively used to sanitize and improve the quality of air in poultry houses. Witkowska and Sowińska [21] and Witkowska et al. [71] demonstrated that fumigation of broiler houses with EOs can improve hygiene standards in poultry farms. In the cited experiments, aqueous solutions of EOs were sprayed in broiler houses. Thyme and peppermint EOs decreased the counts of coliform and Staphylococcus bacteria, molds and yeast-like fungi. The total average counts of aerobic mesophilic bacteria were significantly higher in the control house than in the experimental facility sprayed with EOs. A similar reduction in pathogen counts was noted on walls, in drinkers and feeders. Litter contamination was also reduced in the broiler house fumigated with peppermint EO, but the noted difference was not significant. Both thyme and peppermint EOs reduced bacterial counts, but thyme EO was more effective in eliminating *Enterobacteriaceae*, whereas peppermint EO exerted a stronger inhibitory effect on the proliferation of staphylococci. The cited authors are currently working on establishing safe doses of EOs and their mixtures for sanitizing poultry houses [21,71]. The above results indicate that peppermint and thyme oil mists have no adverse effects on broiler health, peppermint EO could improve performance parameters and EO mists could positively affect the immune system of broilers [72]. The effectiveness of EO mists as health and growth promoters is not well documented in the literature. The cited authors stressed the need for further research in order to identify immune response mechanisms in broilers exposed to different doses of EO mists under real-world conditions.

## 5. Use of Essential Oils in Organic Farming

Due to the growing demand for animal products, there is a need to design new livestock production systems combining food security and sustainability. Organic livestock farming may be a useful strategy to achieve this goal, while meeting consumer expectations regarding animal welfare, health and environmental protection [33,73]. Due to the above mentioned properties [Table 1, Table 2, Table 3 and Table 4], as well as antiparasitic activity [74,75], essential oils might be an emerging strategy in organic livestock farming [32]. Modern agriculture requires the effective control of weeds, diseases and pests in crops. Agrochemicals (herbicides, insecticides, fungicides, bactericides, etc.) are highly effective, but they persist in the environment and can accumulate in food products [76]. Natural plant EOs offer an excellent alternative to synthetic pesticides. They exhibit a broad spectrum of activity against pests, insects and pathogenic fungi, including insecticidal, antifeedant, repellent, oviposition deterrent, growth regulatory and antivector effects [77]. Despite considerable research effort in many laboratories throughout the world and an ever-increasing volume of scientific literature on the pesticidal properties of EOs and their constituents [76,77,78,79,80], the number of commercial biopesticides based on EOs remain surprisingly low [77,81]. According to Pavela and Benelli [81], the existing legislation and authorization procedures need to be simplified to translate research findings into practice. The key challenges facing future biopesticide research include the optimization of EO sources and plant growing conditions, and the development of efficient stabilization processes (e.g., microencapsulation). The advantages of pesticide oil-in-water microemulsions include improved biological efficacy and reduced dosage of pesticides, thus making them a useful strategy in green pesticide technology [77].

## 6. Meat Hygiene and Food Preservation

The studies cited above have confirmed that the quality of meat can be conditioned already during livestock rearing by dietary supplementation with EOs that affect the fatty acid profile of meat and lipid oxidation [6]. Giannenas et al. [65] observed that plant compounds characterized by antioxidant properties can be added to feed to improve the quality of meat during storage.

Essential oils can also be added directly to meat and meat products [6]. Due to their antimicrobial and antioxidant properties, EO additives prevent meat spoilage, and they can be used as effective and, most importantly, natural meat preservatives [34,91]. The addition of EOs to animal-based products can improve their quality and microbiological safety, including both raw and thermally processed meat [16,21,65,71,72].

The addition of EOs serve as natural taste enhancers also affects the taste and aroma of meat products, which improves their sensory attributes and overall acceptability. However, EOs have to be carefully dosed because they can impart an undesirable taste or exert toxic effects at high concentrations, as potent antioxidants [6].

The results of studies investigating the effects of EOs as food additives are presented by many authors [82,83,84,85,86,87,88,89,90]. The analyzed products included proteins from mechanically deboned chicken meat, fresh minced chicken fillet, pure fresh pork fat (lard), minced pork meat, fresh beef meat, ground beef, raw, pasteurized and fermented cow’s milk, milk contaminated with bacteria as well as fish—cod and Atlantic mackerel fillets. The tested EOs exhibited strong antimicrobial activity against pathogenic bacteria [82,83,84,86,88,89]. However, in the case of fermented milk products, the antibacterial effect was associated with reducing the counts of beneficial microbiota [87]. Gómez-Estaca et al. [89] reported that clove EO (followed by rosemary and lavender EOs) exerted the highest inhibitory effect on selected important food pathogens and spoilage bacteria. According to many studies [82,84,85,88,90], EOs can effectively protect food products against lipid oxidation. Oregano EOs have antioxidant properties and are widely used to improve the palatability of meat [92]. In a study by Hać-Szymańczuk and Cegiełka [28], the addition of sage, in particular sage EO, decreased *Enterococcus* counts in pork products. The above authors demonstrated that sage reduced lipid oxidation in pork, and that TBARS (thiobarbituric acid reactive substances formed as a by-product of lipid oxidation) values determined after 5 and 10 days of storage were at least three times lower than in control samples (without the addition of sage). Fasseas et al. [93] reported that the addition of 3% sage EO significantly decreased lipid autoxidation in minced pork and beef, both raw and thermally processed, stored at a temperature of 4 °C for 12 days. Sage EO was a more effective antioxidant in thermally processed than in raw meat. Estevez et al. [94] analyzed the antioxidant effects of sage EO in pork pâté. They found that 0.1% sage EO decreased lipid oxidation and that the natural antioxidant was more effective than its synthetic counterpart.

Essential oils are also added to edible films which can be consumed with food and offer an alternative to conventional food packaging. Edible films are composed of proteins, polysaccharides and EOs. They preserve the quality of food products and prolong their shelf life by inhibiting biological, biochemical and physicochemical changes inside the food matrix. Edible films are enjoying growing popularity, and they could become an environmentally friendly alternative to plastic packaging [92,95]. Vital et al. [92] evaluated the effects of edible coatings made of alginate and rosemary and oregano EOs on the quality, cold storage time and consumer acceptability of beef. The analyzed meat samples were collected from the carcasses of eight young, crossbred bulls. Steaks were randomly divided into four groups: uncoated meat, meat with edible coating, meat with edible coating containing 0.1% rosemary EO, and meat with edible coating containing 0.1% oregano EO. Oregano EO was a more potent antioxidant than rosemary EO, and meat samples with edible coatings containing EOs were characterized by higher antioxidant activity than uncoated samples or samples with coatings without the addition of EOs. Coatings with EOs reduced lipid oxidation in meat, and oregano EO was more effective. All edible coatings inhibited changes in meat color relative to uncoated samples, and they also decreased meat weight loss. Coated samples were characterized by more desirable sensory properties and higher consumer acceptability, and meat with an edible coating containing oregano EO received a higher score in a sensory evaluation [92].

The quality and sensory properties of food products such as beef, chicken and fish, meat and milk also improved after repeated EO treatments [83,86,88,90]. However, in a study by Bonilla et al. [84] the film containing basil and thyme EOs changed the color of meat, which might affect its acceptability by consumers. Similarly, Wrona et al. [85] found that the specific smells of ginger and rose oils applied as active packaging definitely limited their uses to specific types of food that could be regarded as “compatible” with them. Essential oils can extend the shelf life of food products, as confirmed by Ben Jemaa et al. [88] and Karoui et al. [90]. Wrona et al. [85] also found that food packaging with ginger EO and grape seed EO films had a positive effect on meat freshness, extending its shelf life by 6% and 2%, respectively. In contrast, an active film with rose EO exerted a negative influence on the shelf-life of meat by accelerating oxidation. Essential oils used as additives may affect the pH and titratable acidity of food products. In the studies by Gómez-Estaca et al. [89] and Shaltout et al. [86], the pH values of fish meat and beef decreased in response to clove, thyme and cinnamon EOs. Karoui et al. [90] reported that the addition of basil oil decreased the pH of Atlantic mackerel fillets, whereas rosemary oil had no impact on the acidity of fish fillets. In the work of Kostova et al. [87], acid formation was slowed down in samples of cow’s milk containing basil oil, but the noted values of titratable acidity remained within the acceptable ranges.

According to da Silva [91], the development of modern technologies for incorporating EOs into complex food systems in order to minimize sensory changes, enhance their antimicrobial activity and contribute to food quality improvement would be one of the main challenges. Synergistic combinations of different EOs and preservation methods as well as the interactions between the constituents of EOs and food systems should be thoroughly investigated in the future.

Essential oils are sensitive to physicochemical factors such as oxygen, light, temperature and pH. Thus, oxygen in the presence of light leads to the oxidation of unsaturated compounds, accompanied by the formation of free radicals [96]. Poor water solubility also limits EO applications [97]. Novel processing techniques include encapsulation with controlled release of EOs. Polymer microcapsules and nanocapsules loaded with EOs are used in dairy and meat products. Microcapsules slowly release EO components, which ensure that the flavor is preserved, and the shelf life extended [96]. The encapsulation of EOs in zein nanoparticles allows their dispersion in water, which greatly enhances their potential for use in food preservation and the control of human pathogenic bacteria [97].

Encapsulation is also a novel delivery vehicle for animal feed ingredients. Different microencapsulation strategies (oil-carriers can be classified as either polymer-based particles or lipid-based particles) have been proposed to protect the volatile compounds and bioactivity of EOs from degradation and oxidation processes during feed processing and storage, and under different conditions in the gut environment, and to control their release in selected segments of the gut and mixing with the basal feed ingredients [70].

Consumer priorities have become centered on health and healthy eating. Consumers have begun to pay more attention to the contents of their plates, making them more critical in their food choices [98]. The animal-based food industry should strive to meet consumer expectations regarding clean-label products from animals raised organically in line with good welfare practices, including the absence of veterinary drug residues, sustainability, convenience and food safety. This is due to the fact that meat consumption poses a moral dilemma for some consumers who accept that animals are sentient beings. Thus, the feed industry faces a challenge, but also a great opportunity, to improve the sustainability of the production chain [33].

## 7. Summary

Due to their antibacterial and antifungal properties, EOs can inhibit the proliferation of bacteria and fungi in different stages of the food production chain. Cinnamon EO effectively suppressed the growth of many pathogenic bacteria such as *C. perfringens*, *C. botulinum*, *S. aureus*, *E. faecalis*, *S. typhimurium*, *E. coli*, *Y. enterocolitica*, *K. pneumoniae*, *L. monocytogenes*, *P. vulgaris* and *P. aeruginosa*. In turn, *E. coli*, *K. pneumoniae*, *P. aeruginosa* and *S. aureus* were sensitive to all EOs reviewed in this article. According to research, the effectiveness of EOs is largely determined by their concentrations. Tea tree EO is a highly potent antifungal agent that inhibits the growth of most of the analyzed fungal genera and species, including pathogens such as *Microsporum canis*, *T. mentagrophytes* and *T. rubrum*, *Aspergillus* spp., *C. albicans*, *Cryptococcus neoformans*. The reviewed studies also demonstrated that the supplementation of animal diets with EOs delivers health benefits. Thyme, oregano, sage, rosemary and yarrow EOs were found to aid digestion, improve feed intake and the feed conversion ratio, increase the body weight gains of animals and improve other performance parameters, e.g., increase egg weight. Sage, garlic EOs and oils containing cinnamic aldehyde, thymol and carvacrol exert probiotic or immunostimulatory effects. Fumigation of poultry houses with EOs improved hygiene and animal welfare, which suggests that EOs can be used as disinfecting agents to eliminate respiratory pathogens from air. Due to their broad spectrum of activity against pests, insects and pathogenic fungi, including insecticidal, antifeedant, repellent, oviposition deterrent, growth regulatory and antivector effects, EOs may be an alternative to synthetic pesticides in organic farming. Essential oils improve meat quality and hygiene, they effectively preserve food products and prolong their shelf life. These natural preservatives prevent food spoilage and the spread of diseases caused by poor sanitation in food processing. Edible films and coatings containing EOs provide a good barrier against meat spoilage and offer a natural and environmentally-friendly alternative to plastic packaging.

## 8. Conclusions

The use of EOs in different stages of the food processing chain offers a new alternative in animal-based food production. The popularity of EOs is likely to increase in the future because consumers are becoming increasingly aware about the risks associated with food preservatives, the use of antibiotics in animal farming, the presence of antibiotic residues in foods and their contribution to the emergence of antibiotic-resistant bacteria. Essential oils might be an emerging strategy in organic livestock farming and for products acquiring a “clean label”. Oregano, cinnamon, garlic, thyme, black pepper, lavender, peppermint, sage and tea tree EOs deliver considerable benefits in the production of animal-based foods.

However, EOs should be applied with caution because their high concentrations can have toxic effects. Further research is thus needed to establish safe doses of EOs in the food processing industry. A novel delivery technology such as encapsulation with controlled release of EOs needs to be developed to protect them from degradation and oxidation, to minimize undesirable sensory changes, and to enhance their antimicrobial efficacy in feed and food additives.

## Figures and Tables

**Table 1:** Origin, chemical composition and properties of selected essential oils.

Essential Oil	Plant Source	Active Ingredients (% *w*/*v*)	Properties	References
Oregano oil	Oregano (*Origanum vulgare* L.)	thymol (31.5), p-cymene (25.6), carvacrol (16.4)	antibacterial, antifungal, antioxidant, expectorant, relaxant and healing	[10,14,15,16]
Cinnamon oil	Cinnamon (*Cinnamomum zeylanicum, C. cassia, C. inersi*)	cinnamon aldehyde (60–80), eugenol (8–10)	antiseptic, antipyretic, analgesic, anti-inflammatory, digestion and appetite increasing	[17,18]
Garlic oil	Garlic (*Allium sativum* L.)	diallyl disulfide (60), diallyl trisulfide (19–20), allyl methyl trisulfide (15), allyl methyl disulfide (13), diallyl tetrasulfide (8–10)	antibacterial, antifungal, strengthens and regulates immune function	[16,19]
Thyme oil	Thyme (*Thymus vulgaris* L.)	thymol (45–47), p-cymene (32–34), carvacrol (4–5)	antimicrobial, analgesic, antiviral, antifungal, good effect on respiratory diseases, digestion, warming, and appetite increasing	[20,21]
Black pepper oil	Black pepper (*Piper nigrum* L.)	β-caryophyllene (29.9), limonene (13.2), β-pinene (7.9), sabinene (5.9), β-bisabolene (3.9), caryophyllene oxide (3.9)	antiseptic, antibacterial, antifungal, antiprotozoal, antioxidant, anticancer, antipyretic and diuretic	[4,22]
Lavender oil	Narrow-leaved lavender (*Lavandula angustifolia*)	linalyl acetate (25–46), linalool (20–45), eucalyptol (<2.5), terpinen-4-ol (0.1–6.0)	antibacterial, antifungal, immunostimulatory, used in treatment of respiratory disorders and skin diseases	[23,24,25,26]
Peppermint oil	Peppermint (*Mentha x piperita* L.)	menthol (63), p-menthone (19.5)	antioxidant, antibacterial, antiviral, antiallergic, anti-inflammatory, promotes wound healing and inhibits the growth of cancer cells	[4,21,27]
Sage oil	Common sage (*Salvia officinalis* L.)	α-thujone (2–46), camphor (2–46), 1,8-cineole (2–18)	antibacterial, antifungal, antioxidant and anti-inflammatory	[3,15,28,29]
Tea tree oil	Tea tree (*Melaleuca alternifolia*)	terpinen-4-ol (29–45), γ-terpinene (10–28), 1,8-cineole (<15)	antibacterial, antifungal, anti-inflammatory and immunomodulatory	[26,30,31]

**Table 2:** Antibacterial properties of selected essential oils (EOs) and sensitivity of animal pathogenic bacteria to essential oils (minimum inhibitory concentration—MIC).

Taxonomy	Bacterial Species	Pathogenic Effects on Farm Animals	Hosts	Essential Oil ^1^									Unit	References
				Cinnamon	Garlic	Black Pepper	Tea Tree	Lavender	Oregano	Peppermint	Sage	Thyme		
G+ (rod)	*Clostridium perfringens*	necrotizing enterocolitis; enterotoxemia	pigs	+					+	+		+		[3,29,46]
		enterotoxemia; dysentery	lambs											
		necrotic enteritis	chickens											
		hemorrhagic enteritis; enterotoxemia	calves											
G+ (rod)	*Clostridium botulinum*	botulism: toxicosis; paralysis of motor nerves	ruminants, pigs, poultry	+					+			+		[3,17,29,46]
G+ (cocci)	*Staphylococcus aureus*	mastitis; dermatitis; abscesses	ruminants	+	+	+	+	+	+	+	+	+		[3,15,17,20,23,29,36,37,38,40,46]
		mastitis; necrotizing endometritis; abscesses	pigs			>2.0	0.5	1.0	0.12	1.0	1.0	0.25	vol%	
		“bumble foot”—pyogranulomatous lesions of subcutaneous tissue	poultry											
G+ (cocci)	*Enterococcus faecalis*	opportunistic infections (intestinal tract of many animals is a natural environment)	ruminants, pigs, poultry	+		+	+	+	+	+	+	+		[3,15,17,20,29,38,46]
						1.0	2.0	>2.0	0.25	2.0	2.0	0.5	µL/mL	
G- (rod)	*Salmonella typhimurium*	food poisoning; enteritis; septicemia	ruminants, pigs, poultry	+		+	+	+	+	+	+	+		[3,15,17,29,35,38,39,46]
						>2.0	0.5	>2.0	0.12	1.0	2.0	>2.0	vol%	
G- (rod)	*Escherichia coli*	food poisoning; septicemia; coliform mastitis;	ruminants											[3,15,17,20,23,29,36,37,38,39,46]
		piglet meningitis; weanling enteritis; oedema; mastitis-metritis-agalactia (MMA) syndrome	pigs	+	+	+	+	+	+	+	+	+		
		omphalitis; colisepticemia; coligranuloma	poultry			>2.0	0.25	0.5	0.12	0.5	0.5	0.12	vol%	
G- (rod	*Yersinia enterocolitica*	latent infections with sporadic cases of enteritis or generalized infections	ruminants, pigs, poultry	+					+			+		[3,15,29,40,46]
												1.0	µL/mL	
G- (rod)	*Klebsiella pneumoniae*	pneumonia; urinary tract infections, sepsis; mastitis	cattle	+	+	+	+	+	+	+	+	+		[3,17,20,29,38,46]
						>2.0	0.5	2.0	0.12	1.0	2.0	0.25	vol%	
G- (rod)	*Listeria monocytogenes*	septicemia; necrotic foci in liver and other organs; central nervous system infections; abortion; iritis; mastitis	ruminants	+					+	+	+	+		[3,15,29,40,46]
											3–5.0	2.0	µL/mL	
G- (rod)	*Pseudomonas aeruginosa*	mastitis; skin, uterine and respiratory infections; enteritis; arthritis;	ruminants											[3,17,20,23,29,37,38,46]
		respiratory infections; enteritis; otitis	pigs	+	+	+	+	+	+	+	+	+		
		septicemia	poultry			>2.0	>2.0	>2.0	>2.0	>2.0	>2.0		vol%	

^1^ + EOs with antibacterial effects.

**Table 3:** Antifungal properties of selected essential oils (EOs) and sensitivity of animal pathogenic fungi to essential oils (minimum inhibitory concentration—MIC).

Taxonomy	Fungal Genus/Species	Pathogenic Effects on Farm Animals	Hosts	Essential Oil ^1^									Unit	References
				Cinnamon	Garlic	Black Pepper	Tea Tree	Lavender	Oregano	Peppermint	Sage	Thyme		
Mold	*Trichophyton* spp.	dermatomycoses: skin, nails, claws and hair lesions (ringworm)	ruminants, pigs, poultry		+		+	+	+		+	+		[23,29,41,43,45,46]
								1.0			1.0	0.25	vol%	
Mold	*Microsporum* spp.						+	+	+		+	+		
								0.25			0.25	0.125	vol%	
Mold	*Aspergillus* spp.	mycotic abortion; mastitis; intestinal aspergillosis	cattle	+			+	+	+		+	+		[15,23,29,37,41,42,43,44]
		pneumonia and air sacculitis; mycotoxicosis	poultry					1.0	0.025		0.5	0.25	vol%	
Mold	*Penicillium* spp.	mycotoxicoses	ruminants, pigs, poultry				+	+	+		+	+		[15,29,43,44,45,46]
								0.5	0.025		0.5	0.25	vol%	
Mold	*Fusarium* spp.							+		+	+	+		[3,15,29,43,46]
										0.016		0.125	vol%	
Mold	*Alternaria alternata*						+	+			+	+		[15,29,43,45]
								0.5			0.25	0.5	vol%	
Mold	*Cladosporium* spp.						+	+			+	+		[29,43]
								0.25			0.125	0.125	vol%	
Mold	*Rhizopus oryzae*	pulmonary disease; intestinal infection	cattle, pigs						+					[15,46]
Mold	*Malassezia sympodialis*	otitis	cattle				+							[29,46]
Yeast	*Candida albicans*	pneumonic, enteric and generalized candidiasis (gastro-esophageal ulcers, rumenitis); mastitis	cattle											[29,37,38,41,42,46]
		gastro-esophageal ulcers	pigs	+		+	+	+	+	+	+	+		
		mouth or esophagus mycosis causing stunting and high mortality in young birds	poultry			>2.0	0.5	0.5	0.12		0.5	0.12	vol%	
Yeast	*Cryptococcus neoformans*	rare cause of mastitis; respiratory infection with frequent dissemination to CNS	cattle	+			+					+		[29,41,46]

^1^ + EOs with antifungal effects.

**Table 4:** The effect of essential oils (EOs) as functional feed ingredients on animal performance and product quality characteristics.

Feed/Water Additive		Species	Performance Parameters ^1−6^	Problems and Limitation	References
Essential Oil/Active Ingredient	Dose				
carvacrol, cinnamaldehyde, paprika, oleoresin	271 mg/kg of feed	pigs	+ protect pig’s blood lymphocytes against oxidative DNA damage	− strong taste and smell; − can affect the function of intestinal microflora, cause allergies, suppress feed intake and can be stored in tissues; − not sufficient to fully prevent lipid peroxidation induced by high intake of lightly oxidizable PUFA	[47]
carvacrol, cinnamaldehyde, paprika, oleoresin	5% carvacrol, 3% cinnamaldehyde 2% capsicum and oleoresin	pigs	+ probiotic effect	− more specific studies are required to clarify how these products modify pig gastrointestinal bacteria, which would facilitate their most judicious use in field conditions	[48]
clove, rosemary EOs and protected eugenol, thymol, vanillin	2–4 g/animal/d of EOs, 2 g/animal/d of protected oils	heifers	+ BWG and FCR	− use of rosemary EO on its own tended to decrease animal performance	[49]
EOs product based on eugenol and cinnamaldehyde	525 mg/animal/d	dairy cows	⚬ moderate effects on ruminal fermentation; + ruminal isobutyrate concentration and total-tract digestibility of neutral-detergent fiber; ⚬ milk production or composition;	− the true value of EOs for altering rumen microbial fermentation and animal production must be assessed *in vivo*; − the EO product increased cumulative ammonia emission from manure	[50]
thyme, sage, rosemary EOs	200 mg of each oil/kg of feed	laying hens	− ratio of triglyceride and cholesterol of serum, + egg weight; ⚬ egg white; − egg yolk; + eggshell; + FCR	− discrepancy of results concerning the addition of EOs to a layer diet on egg production, FI and FCR	[51]
oregano EO	50–100 mg/kg of feed	laying hens	⚬ egg production, shape, weight, yolk diameter, height and color; + antioxidative activity; ⚬ FCR, FI and BWG	− additional research is needed towards developing methods for determination of antioxidant constituents of oregano EO passed into egg yolk	[52]
carvacrol	the diet contained 5% corn oil and 200 ppm of carvacrol	broiler chickens	− plasma triglycerides and phospholipids; ⚬ total and HDL cholesterol; − FCR, FI and BWG	− possibly, carvacrol negatively affected feed intake by modulating appetite	[53]
protected blend of EO (ginger oil and carvacrol)	blend of EOs (1%) at 1.5 g/kg of feed	broiler chickens	+ bird’s resistance to necrotic enteritis (NE); + increase villus length and villus length: crypt depth ratio; + BWG	− *in vivo* studies have to be conducted to evaluate precisely the effect of EOs in poultry NE	[54]
carvacrol, cinnamaldehyde	100 mg/kg of feed	broiler chickens	+ probiotic effects; + the total amino acid digestibility; + FCR; ⚬ BWG	− difficult standardization	[55]
blend of EOs containing thymol and cinnamic aldehyde	15 g/metric ton thymol and 5 g/metric ton cinnamaldehyde	broiler chickens	+ probiotic effects; effects on cecal metabolites; FCR, FI and BWG	− the performance effects are likely to be dependent on the quality and quantity of EOs; the variation in responses may also be associated with the type of EO present in the blend and their potential synergistic, additive or counteractive effects	[56]
peppermint, eucalyptus EOs	0.25 mL/l of drinking water	broiler chickens	+ activation of immune organs; + immunomodulatory effect on innate-cell mediated and humoral immune response against the Newcastle Disease virus; + FCR and BWG	− most studies investigated blends of various active compounds and reported their effects on production performance rather than the physiological impacts	[57,58]
volatile oil mixtures of oregano, rosemary, fennel	400 mg/kg of feed	broiler chickens	+ stimulate the growth; + improve the intestinal microbial balance (reduction of coliform bacteria and an increase in *Lactobacillus* spp. counts)	− possible synergism with different VOs may promote synergistic or antagonistic effects of bioactive compounds, thus investigating the combinations of VOs, is of far higher importance than investigating the effects of each VO in isolation	[59]
oregano, thyme, rosemary, yarrow EOs	1g of each oil/kg of feed	broiler chickens	+ thyme and yarrow had the greater effect on growth; − oregano and rosemary reduced BWG and FI; ⚬ probiotic effect; ⚬ nutrient digestibility	− although the mechanisms behind terpene interactions are unknown, synergistic or antagonistic interactions in a plant extract may affect its antimicrobial potential; − the form of herbal supplementation may also be influential in determining bioactivity	[60]
thymol, carvacrol	60, 100, 200 mg/kg of feed (1:1 thymol:carvacrol)	broiler chickens	+ antioxidant and digestive enzyme activities; + immune response; + FCR, FI and BWG	− the results of the effects of thymol and carvacrol on growth performance in poultry were not consistent	[61]
thyme EO	50–100 mg/kg of feed	quails	− abdominal fat weight and percentage; − intestinal pH at the end of experiment; + FCR, FI and BWG	− active components of plant oils may vary because different methods of extraction and could have different effects on the activity	[62]
blend of microencapsulated garlic, carvacrol, and thymol synthetic EOs	diet supplemented with 0.5% of the EOs functional additive	fishes (*Sparus aurata*)	+ the modulation of the intestine transcriptional immune profile; + microbiota composition ⚬ BWG	− exact mechanisms are still elusive	[63]

^1^ + improve, ^2^ − decrease, ^3^ o no effect, ^4^ FCR—feed conversion ratio, ^5^ FI—feed intake/consumption, ^6^ BWG—body weight gains.

**Table 5:** The effect of essential oils (EOs) and their active ingredients on the animal-based food products.

Animal-Based Food Product	Essential Oil/Active Ingredient	Effects	Problems and Limitations	References
mechanically deboned chicken meat protein	thyme, clove and rosemary EO (1.5%) films	+ antimicrobial *activity against B. subtilis, S. aureus, E. coli* (clove), *L. monocytogenes* (thyme); + antioxidant activity	⚬ slightly yellowish color of films; ⚬ a rough surface of rosemary EO film	[82]
fresh minced chicken fillet	rosemary and *Nigella sativa* EOs (0.1–0.5%)	+ antimicrobial activity against *S. aureus* (*Nigella sativa* oil was more effective); + the sensory characteristics (*Nigella sativa* oil)	− the EOs may be selected for use as potential food biopreservatives in foods, depending upon the desired flavor of the products	[83]
pure fresh pork fat (lard), minced pork meat	chitosan films containing basil or thyme EOs (0.5–1%)	+ protect pork fat from oxidation (oxygen permeability in films after inclusion of EOs); ⚬ antimicrobial activity	− changing the color of meat can affect its acceptability	[84]
fresh beef meat	ginger, grape seed and rose films with EOs (25–50% in active masterbatch)	+ antioxidant properties; + shelf life (ginger, grape seed); − shelf life (rose)	− very strong and characteristic smell of EOs from ginger and rose, causes their limitation in food packaging	[85]
ground beef	thyme (1–2%) and cinnamon (0.5–1.5%) EOs	+ antimicrobial activity against *Enterobacteriaceae* and coliform bacteria; + sensory properties (higher concentration was more effective); − pH	− in low concentrations the oils may be less effective in meet quality and shelf life	[86]
raw, pasteurized and fermented cow milk	basil EO (0,8 mg/kg of final product)	− growth of *Lactobacillus delbrueckii subsp. bulgaricus* (for fermented milk products this is a negative result); − lactic acid formation (the titratable acidity value was within the standards)	− essential oils may slow down the growth of the desired bacterial culture in fermented milk products	[87]
contaminated milk by bacteria	thyme EO (7%) encapsulated into oil in water nanoemulsion	+ significant inhibition of all bacterial populations (*S. aureus, Bacillus licheniformis, Enterococcus hirae*); + oxidative and fermentation stability; + milk quality; + shelf life	− the strong aroma and flavor can affect consumer acceptance	[88]
fish—cod fillets	Gelatin-chitosan film containing clove EO (10 µL of EO/ 10 µL of fish)	+ antimicrobial activity (very pronounced inhibition for H_2_S producing bacteria); − pH (below 7); − the occurrence of total volatile nitrogen; + shelf life	− the use of EOs in foods could be limited because they would confer very different flavors and smells from those natural to the food in question, as in the case of fish	[89]
Fish—Atlantic mackerel fillets	rosemary and basil EOs	− lipid peroxidation; + to some extent preserved the appearance of fresh fillets; + shelf life; ⚬ pH (rosemary oil), (-) pH (basil oil)	− some of the results were inconsistent with other authors’ findings	[90]

^1^ + improve, ^2^ − decrease, ^3^ o no effect.

## Data Availability

Not applicable.

## References

[1] Baser K.H.C., Buchbauer G., Baser K.H.C., Buchbauer G. (2009). Introduction. Handbook of Essential Oils and Science, Technology and Applications.

[2] Franz C., Novak J., Baser K.H.C., Buchbauer G. (2009). Sources of essential oils. Handbook of Essential Oils and Science, Technology and Applications.

[3] Sienkiewicz M., Denys P., Kowalczyk E. (2011). Antibacterial and immunostimulatory effect of essential oils. Int. Rev. Allergol. Clin. Immunol..

[4] Falleh H., Ben Jemaa M., Saada M., Ksouri R. (2020). Essential oils: A promising eco-friendly food preservative. Food Chem..

[5] Zdrojewicz Z., Minczakowska K., Klepacki K. (2014). The role of aromatherapy in medicine. Fam. Med. Prim. Care Rev..

[6] Zhai H., Liu H., Wang S., Wu J., Kluenter A.M. (2018). Potential of essential oils for poultry and pigs. Anim. Nutr..

[7] Benchaar C., Calsamiglia S., Chaves A.V., Fraser G.R., Colombatto D., McAllister T.A., Beauchemin K.A. (2008). A review of plant-derived essential oils in ruminant nutrition and production. Anim. Feed Sci. Technol..

[8] European Parliament, European Council (2003). Regulation (EC) No 1831/2003 on the European Parliament and of the Council of 22 September 2003. On Additives for Use in Animal Nutrition. Off. J. Eur. Union.

[9] Przeniosło-Siwczyńska M., Kwiatek K. (2013). Why the use of antibiotic growth promoters in animal feeds was banned?. Życie Wet..

[10] Souza E.L., Stamford T.L.M., Lima E.O., Trajano V.N. (2007). Effectiveness of *Origanum vulgare* L. essential oil to inhibit the growth of food spoiling yeasts. Food Control..

[11] Cegiełka A. (2020). ‘Clean label’ as one of the leading trends in the meat industry in the world and in Poland—A review. Rocz. Panstw. Zakl. Hig..

[12] Wójcik W., Solarczyk P., Łukasiewicz M., Puppel K., Kuczyńska B. (2018). Trends in animal production from organic farming [review]. Acta Innov..

[13] Naeem A., Abbas T., Ali T.M., Hasnain A. (2018). Essential oils: Brief background and uses. Ann. Short Rep..

[14] Hać-Szymańczuk E., Lipińska E., Grzegrzółka O. (2012). Estimation of the antibacterial activity of the oregano (*Origanum vulgare* L.). Bromat. Chem. Toksykol..

[15] Hać-Szymańczuk E., Lipińska E., Chlebowska-Śmigiel A. (2014). Comparison of antimicrobial activity of sage (*Salvia officinalis* L.) And oregano (*Origanum vulgare* L.) Essential oils. Zesz. Probl. Post. Nauk Roln..

[16] Kirkpinar F., Ünlü H.B., Serdaroğlu M., Turp G.Y. (2014). Effects of dietary oregano and garlic essential oils on carcass characteristics, meat composition, colour, pH and sensory quality of broiler meat. Br. Poult. Sci..

[17] Kędzia A. (2011). The activity of cinnamon oil (*Oleum cinnamoni*) against anaerobic bacteria. Post. Fitoter..

[18] Kaławaj K., Lemieszek M.K. (2015). Health promoting properties of cinnamon. Med. Ogólna Nauki Zdr..

[19] Kędzia A. (2009). Garlic oil—Chemical components, pharmacological and medical activity. Post. Fitoter..

[20] Kędzia A., Dera-Tomaszewska B., Ziółkowska-Klinkosz M., Kędzia A.W., Kochańska B., Gębska A. (2012). Activity of thyme oil (*Oleum thymi*) against aerobic bacteria. Post. Fitoter..

[21] Witkowska D., Sowińska J. (2013). The effectiveness of peppermint and thyme essential oil mist in reducing bacterial contamination in broiler houses. Poult. Sci..

[22] Kozłowska-Lewecka M., Wesołowski W., Borowiecka J. (2011). Analysis of contents of essential oils in white and black pepper determined by GC/MS. Bromatol. Chem. Toksyk..

[23] Cavanagh H.M.A., Wilkinson J.M. (2005). Lavender essential oil: A review. Aust. Infect. Control.

[24] Adaszyńska-Skwirzyńska M., Swarcewicz M. (2014). Chemical composition and biological activity of medical lavender. Wiad. Chem..

[25] Kraśniewska K., Gniewosz M., Kosakowska O., Pobiega K. (2017). Chemical composition and antimicrobial properties of essential oil from lavender (*Lavandula angustifolia* L.) in commercial available preparation. Post. Fitoter..

[26] Sandner G., Heckmann M., Weghuber J. (2020). Immunomodulatory activities of selected essential oils. Biomolecules.

[27] Łyczko J., Piotrowski K., Kolasa K., Galek R., Szumny A. (2020). *Mentha piperita* L. micropropagation and the potential influence of plant growth regulators on volatile organic compound composition. Molecules.

[28] Hać-Szymańczuk E., Cegiełka A. (2015). Evaluation of antimicrobial and antioxidant activity of sage in meat product. Żywn. Nauka Technol. Jakość.

[29] Swamy M.K., Akhtar M.S., Sinniah U.R. (2016). Antimicrobial properties of plant essential oils against human pathogens and their mode of action: An updated review. Evid. Based Complement. Alternat. Med..

[30] Wyszkowska-Kolatko M., Koczurkiewicz P., Pękala E. (2016). Cytotoxic effect of tea tree oil—*In vitro* studies. Post. Fitoter..

[31] Taiwo M.O., Adebayo O.S. (2017). Plant essential oil: An alternative to emerging multidrug resistant pathogens. J. Microbiol. Exp..

[32] Franz C., Baser K.H.C., Windisch W. (2010). Essential oils and aromatic plants in animal feeding—A European perspective. A review. Flavour Fragr. J..

[33] Escribano E.J. (2018). Organic feed: A bottleneck for the development of the livestock sector and its transition to sustainability?. Sustainability.

[34] Nehme R., Andrés S., Pereira R.B., Ben Jemaa M., Bouhallab S., Ceciliani F., López S., Rahali F.Z., Ksouri R., Pereira D.M. (2021). Essential oils in livestock: From health to food quality. Antioxidants.

[35] Osek J., Wieczorek K. (2020). Zoonoses in humans and presence of their etiological agents in animals and in food in the European Union Member States in 2018. Życie Wet..

[36] Inouye S., Takizawa T., Yamaguchi H. (2001). Antibacterial activity of essential oils and their major constituents against respiratory tract pathogens by gaseous contact. J. Antimicrob. Chemother..

[37] Sharifi-Rad J., Sureda A., Tenore G.C., Daglia M., Sharifi-Rad M., Valussi M., Tundis R., Sharifi-Rad M., Loizzo M.R., Ademiluyi A.O. (2017). Biological activities of essential oils: From plant chemoecology to traditional healing systems. Molecules.

[38] Hammer K.A., Carson C.F., Riley T.V. (1999). Antimicrobial activity of essential oils and other plant extracts. J. Appl. Microbiol..

[39] Tanu B., Harpreet K. (2016). Benefits of essential oil. J. Chem. Pharm. Res..

[40] Rota C., Carraminana J.J., Burillo J., Herrera A. (2004). *In vitro* antimicrobial activity of essential oils from aromatic plants against selected foodborne pathogens. J. Food Prot..

[41] Sienkiewicz M., Denys A. (2008). Activity of essential oils in prevention and therapy of mycoses. Pediatr. Med. Rodz..

[42] Laranjo M., Fernandez-Leon A.M., Potes M.E., Agulheiro-Santos A.C., Elias M., Mendez-Vilas A. (2017). Use of essential oils in food preservation. Antimicrobial Research: Novel Bioknowledge and Educational Programs.

[43] Tullio V., Nostro A., Mandras N., Dugo P., Banche G., Cannatelli M.A., Cuffini A.M., Alonzo V., Carlone N.A. (2007). Antifungal activity of essential oils against filamentous fungi determined by broth microdilution and vapour contact methods. J. Appl. Microbiol..

[44] Puškárová A., Bučková M., Kraková L., Pangallo D., Kozics K. (2017). The antibacterial and antifungal activity of six essential oils and their cyto/genotoxicity to human HEL 12469 cells. Sci. Rep..

[45] Stupar M., Grbić M.L., Džamić A., Unković N., Ristić M., Jelikić A., Vukojević J. (2014). Antifungal activity of selected essential oils and biocide benzalkonium chloride against the fungi isolated from cultural heritage objects. S. Afr. J. Bot..

[46] Markey B., Leonard F., Archambault M., Cullinane A., Maguire D. (2013). Clinical Veterinary Microbiology.

[47] Frankič-Korošec T., Voljč M., Salobir J., Rezar V. (2009). Use of herbs and spices and their extracts in animal nutrition. Acta Agric. Slov..

[48] Castillo M., Martin-Orue S.M., Roca M., Manzanilla E.G., Badiola I., Perez J.F., Gasa J. (2006). The response of gastrointestinal microbiota to avilamycin, butyrate, and plant extracts in early-weaned pigs. J. Anim. Sci..

[49] De Souza K.A., de Oliveira Monteschio J., Mottin C., Ramos T.R., de Moraes Pinto L.A., Eiras C.E., Guerrero A., do Prado I.N. (2019). Effects of diet supplementation with clove and rosemary essential oils and protected oils (eugenol, thymol and vanillin) on animal performance, carcass characteristics, digestibility, and ingestive behavior activities for Nellore heifers finished in feedlot. Livest. Sci..

[50] Tekippe J.A., Tacoma R., Hristov A.N., Lee C., Oh J., Heyler K.S., Cassidy T.W., Varga G.A., Bravo D. (2013). Effect of essential oils on ruminal fermentation and lactation performance of dairy cows. J. Dairy Sci..

[51] Bölükbaşı Ş.C., Erhan M.K., Kaynar Ö. (2008). The effect of feeding thyme, sage and rosemary oil on laying hen performance, cholesterol and some proteins ratio of egg yolk and *Escherichia coli* count in feces. Europ. Poult. Sci..

[52] Florou-Paneri P., Nikolakakis I., Giannenas I., Koidis A., Botsoglou E., Dotas V., Mitsopoulos I. (2005). Hen performance and egg quality as affected by dietary oregano essential oil and alpha-tocopheryl acetate supplementation. Int. J. Poult. Sci..

[53] Lee K.W., Everts H., Kappert H.J., Yeom K.H., Beynen A.C. (2003). Dietary carvacrol lowers body weight gain but improves feed conversion in female broiler chickens. J. Appl. Poult. Res..

[54] Jerzsele A., Szeker K., Csizinszky R., Gere E., Jakab C., Mallo J.J., Galfi P. (2012). Efficacy of protected sodium butyrate, a protected blend of essential oils, their combination, and *Bacillus amyloliquefaciens* spore suspension against artificially induced necrotic enteritis in broilers. Poult. Sci..

[55] Jamroz D., Wiliczkiewicz A., Wertelecki T., Orda J., Skorupińska J. (2005). Use of active substances of plant origin in chicken diets based on maize and locally grown cereals. Br. Poult. Sci..

[56] Tiihonen K., Kettunen H., Bento M.H.L., Saarinen M., Lahtinen S., Ouwehand A.C., Schulze H., Rautonen N. (2010). The effect of feeding essential oils on broiler performance and gut microbiota. Br. Poult. Sci..

[57] Awaad M.H.H., Abdel-Alim G.A., Sayed Kawkab K.S.S., Ahmed A., Nada A.A., Metwalli A.S.Z., Alkhalaf A.N. (2010). Immunostimulant effects of essential oils of peppermint and eucalyptus in chickens. Pak. Vet. J..

[58] Awaad M.H.H., Afify M.A.A., Zoulfekar S.A., Mohammed F.F., Elmenawy M.A., Hafez H.M. (2016). Modulating effect of peppermint and eucalyptus essential oils on vVND infected chickens. Pak. Vet. J..

[59] Cetin E., Yibar A., Yesilbag D., Cetin I., Cengiz S.S. (2016). The effect of volatile oil mixtures on the performance and ilio-caecal microflora of broiler chickens. Br. Poult. Sci..

[60] Cross D.E., McDevitt R.M., Hillman K., Acamovic T. (2007). The effect of herbs and their associated essential oils on performance, dietary digestibility and gut microflora in chickens from 7 to 28 days of age. Br. Poult. Sci..

[61] Hashemipour H., Kermanshahi H., Golian A., Veldkamp T. (2013). Effect of thymol and carvacrol feed supplementation on performance, antioxidant enzyme activities, fatty acid composition, digestive enzyme activities, and immune response in broiler chickens. Poult. Sci..

[62] Denli M., Okan F., Uluocak A.N. (2004). Effect of dietary supplementation of herb essential oils on the growth performance, carcass and intestinal characteristic of quail. S. Afr. J. Anim. Sci..

[63] Firmino J.P., Vallejos-Vidal E., Balebona M.C., Ramayo-Caldas Y., Cerezo I.M., Salomón R., Tort L., Estevez A., Moriñigo M.Á., Reyes-López F.E. (2021). Diet, immunity, and microbiota interactions: An integrative analysis of the intestine transcriptional response and microbiota modulation in gilthead seabream (*Sparus aurata*) fed an essential oils-based functional diet. Front. Immunol..

[64] Skomorucha I., Sosnówka-Czajka E. (2012). Effects of dietary herbal supplements on poultry productivity and health. Wiad. Zoot..

[65] Giannenas I., Bonos E., Christaki E., Florou-Paneri P. (2013). Essential oils and their applications in animal nutrition. Med. Aromat. Plants.

[66] Belanche A., Newbold C.J., Morgavi D.P., Bach A., Zweifel B., Yáñez-Ruiz D.R. (2020). A Meta-analysis describing the effects of the essential oils blend Agolin Ruminant on performance, rumen fermentation and methane emissions in dairy cows. Animals.

[67] IRTA (2015). Review of Immune Stimulator Substances/Agents That Are Susceptible of Being Used as Feed Additives: Mode of Action and Identification of End-Points for Efficacy Assessment.

[68] Lippens M., Huyghebaert G., Cerchiari E. (2005). Effect of the use of coated plant extracts and organic acids as alternatives for antimicrobial growth promoters on the performance of broiler chickens. Eur. Poult. Sci..

[69] Da Silva B.D., Campos Bernardes P., Fontes Pinheiro P., Fantuzzi E., Consuelo D.R. (2021). Chemical composition, extraction sources and action mechanisms of essential oils: Natural preservative and limitations of use in meat products. Meat Sci..

[70] Stevanović Z.D., Bošnjak-Neumüller J., Pajić-Lijaković I., Raj J. (2018). Essential oils as feed additives—Future perspectives. Molecules.

[71] Witkowska D., Sowińska J., Żebrowska J., Mituniewicz E. (2016). The antifungal properties of peppermint and thyme essential oils misted in broiler houses. Braz. J. Poult. Sci..

[72] Witkowska D., Sowińska J., Murawska D., Matusevicius P., Kwiatkowska-Stenzel A., Mituniewicz T., Wójcik A. (2019). Effect of peppermint and thyme essential oil mist on performance and physiological parameters in broiler chickens. S. Afr. J. Anim. Sci..

[73] Escribano A.J., Konvalina P. (2016). Organic livestock farming—challenges, perspectives, and strategies to increase its contribution to the agrifood system’s sustainability—A review. Organic Farming—A Promising Way of Food Production.

[74] Mohiti-Asli M., Ghanaatparast-Rashti M. (2015). Dietary oregano essential oil alleviates experimentally induced coccidiosis in broilers. Prev. Vet. Med..

[75] Bozkurt M., Ege G., Aysul N., Aksit H., Tuzun A.E., Kucukyılmaz K., Borum A.E., Uygun M., Aksit D., Aypak S. (2016). Effect of anticoccidial monensin with oregano essential oil on broilers experimentally challenged with mixed *Eimeria* spp.. Poult. Sci..

[76] Durán-Lara E.F., Valderrama A., Marcian A. (2020). Natural organic compounds for application in organic farming. Agriculture.

[77] Mohan M., Haider S.Z., Andola H.C., Purohit V.K. (2011). Essential oils as green pesticides: For sustainable agriculture. Res. J. Pharm. Biol. Chem. Sci..

[78] Campiglia E., Mancinelli R., Cavalieri A., Caporali F. (2007). Use of essential oils of cinnamon, lavender and peppermint for weed control. Ital. J. Agron..

[79] Robu V., Covaci G., Popescu I.M. (2015). The use of essential oils in organic farming. Res. J. Agric. Sci..

[80] Frabboni L., Tarantino A., Petruzzi F., Disciglio G. (2019). Bio-herbicidal effects of oregano and rosemary essential oils on chamomile (*Matricaria chamomilla* L.) crop in organic farming system. Agronomy.

[81] Pavela R., Benelli G. (2016). Essential oils as ecofriendly biopesticides? Challenges and constraints. Trends Plant Sci..

[82] Sarıcaoglu F.T., Turhan S. (2020). Physicochemical, antioxidant and antimicrobial properties of mechanically deboned chicken meat protein films enriched with various essential oils. Food Packag. Shelf Life.

[83] Ibrahim H.M., Hassan M.A., Amin R.A., Shawqy N.A., Elkoly R.L. (2018). Effect of some essential oils on the bacteriological quality of some chicken meat products. J. Benha Vet. Med..

[84] Bonilla J., Vargas M., Atarés L., Chiralt A. (2014). Effect of chitosan essential oil films on the storage-keeping quality of pork meat products. Food Bioprocess Tech..

[85] Wrona M., Silva F., Salafranca J., Nerina C., Alfonso M.J., Caballero M.A. (2021). Design of new natural antioxidant active packaging: Screening flowsheet from pure essential oils and vegetable oils to ex vivo testing in meat samples. Food Cont..

[86] Shaltout F.A., Thabet M.G., Koura H.A. (2017). Impact of some essential oils on the quality aspect and shelf life of meat. J. Nutr. Food Sci..

[87] Kostova I., Damyanova S.T., Ivanova N., Stoyanova A., Ivanova M., Vlaseva R. (2016). Use of essential oils in dairy products. Essential oil of basil (*Ocimum basilicum* L.). Indian J. Appl. Res..

[88] Ben Jemaa M., Falleh H., Neves M.A., Isoda H., Nakajima M., Ksouri R. (2017). Quality preservation of deliberately contaminated milk using thyme free and nanoemulsified essential oils. Food Chem..

[89] Gómez-Estaca J., López de Lacey A., López-Caballero M.E., Gómez-Guillén M.C., Montero P. (2010). Biodegradable gelatin-chitosan films incorporated with essential oils as antimicrobial agents for fish preservation. Food Microbiol..

[90] Karoui R., Hassoun A. (2017). Efficiency of rosemary and basil essential oils on the shelf-life extension of Atlantic mackerel (*Scomber scombrus*) fillets stored at 2 °C. J. AOAC Int..

[91] Silva R.S., Lima A.S., da Silva L.P., Silva R.N., Pereira E.M., de Oliveira F.L.N., Azerêdo G.A. (2019). Addition of essential oils and inulin for production of reduced salt and fat ham. Aust. J. Crop Sci..

[92] Vital A.C.P., Guerrero A., de Oliveira Monteschio J., Valero M.V., Carvalho C.B., de Abreu Filho B.A., Madrona G.S., do Prado I.N. (2016). Effect of edible and active coating (with rosemary and oregano essential oils) on beef characteristics and consumer acceptability. PLoS ONE.

[93] Fasseas M.K., Mountzouris K.C., Tarantilis P.A., Polissiou M., Zervas G. (2007). Antioxidant activity in meat treated with oregano and sage essential oils. Food Chem..

[94] Estevez M., Ramirez M., Ventanas S., Cava R. (2007). Sage and rosemary essential oils versus BHT for inhibition of lipid oxidative reactions in liver pâté. LWT Food Sci. Technol..

[95] Chlebowska-Śmigiel A., Hać-Szymańczuk E., Gniewosz M. (2014). The influence of edible coating on microbiological changes in beef meat under refrigeration. Zesz. Probl. Post. Nauk Roln..

[96] Dima C., Dima S. (2015). Essential oils in foods: Extraction, stabilization, and toxicity. Curr. Opin. Food Sci..

[97] Wu Y., Luo Y., Wang Q. (2012). Antioxidant and antimicrobial properties of essential oils encapsulated in zein nanoparticles prepared by liquid-liquid dispersion method. LWT Food Sci. Technol..

[98] Sousa I., Raymundo A., Torres M.D. (2020). Eco-novel food and feed. Appl. Sci..

